# Eight-day fasting modulates serum kynurenines in healthy men at rest and after exercise

**DOI:** 10.3389/fendo.2024.1403491

**Published:** 2024-06-12

**Authors:** Ulana Juhas, Joanna Reczkowicz, Jakub Antoni Kortas, Małgorzata Żychowska, Karol Pilis, Ewa Ziemann, Inga Cytrych, Jędrzej Antosiewicz, Andżelika Borkowska

**Affiliations:** ^1^ Department of Bioenergetics and Physiology of Exercise, Medical University of Gdańsk, Gdańsk, Poland; ^2^ Department of Health and Life Sciences, Gdańsk University of Physical Education and Sport, Gdańsk, Poland; ^3^ Department of Biological Foundations of Physical Culture, Kazimierz Wielki University, Bydgoszcz, Poland; ^4^ Department of Health Sciences, Jan Długosz University in Częstochowa, Częstochowa, Poland; ^5^ Department of Athletics, Strength and Conditioning, Poznan University of Physical Education, Poznań, Poland; ^6^ Masdiag Sp. z o.o., Warsaw, Poland

**Keywords:** kynurenic acid, picolinic acid, xanthurenic acid, Phe/Tyr ratio, 3-hydroxyanthranilic acid

## Abstract

**Introduction:**

Tryptophan’s (Trp) metabolites are undervalued markers of human health. Their serum concentrations are modified by physical exercise and other factors, among which fasting has a well-documented role. Although this mechanism is hardly explored, thus, the study aimed to determine the effect of the 8-day fasting period and the impact of such a procedure on a single bout of an endurance exercise on the concentration of kynurenine pathway (KP) metabolites.

**Methods:**

10 participants fasted for 8 days, and 10 as a control group participated in the study. The exercise was performed at baseline after an overnight fast and repeated post 8 days.

**Results:**

The 8 days of fasting increased the resting 3-hydroxy-L-kynurenine (3HK), picolinic acid (PA), kynurenic acid (KYNA), and xanthurenic acid (XA) serum concentration. Also elevated phenylalanine (Phe) and tyrosine (Tyr) levels were recorded, suggesting expanded proteolysis of muscle proteins. In turn, physical activity caused a decrease in the concentration of 3-hydroxyanthranilic acid (3HAA) and PA after fasting. The obtained results were not recorded in controls.

**Conclusion:**

The results of this study show that the health-promoting effects of fasting are associated with changes in the KYN pathway. The increase in the concentration of PA and XA metabolites following fasting is capable of penetrating the blood-brain barrier, and KYNA, which initiates several beneficial changes, supports this assumption.

## Introduction

Tryptophan (Trp) is an essential amino acid that is degraded primarily via the kynurenine pathway (KP), and it generates metabolites referred to as kynurenines (1). Dysregulation of Trp and KP has been linked to the pathogenesis and progression of various diseases, including type 2 diabetes, pulmonary arterial hypertension, neurodegeneration, depression, dementia, inflammation, and some others (2–5). In some disorders, increased concentrations of kynurenine (KYN), KYN/tryptophan (Trp) ratio, and kynurenic acid (KYNA) to 3-hydroxy kynurenine (3HK) ratio are observed (6). Metabolites of KP exert various physiological functions by modifying energetic metabolism, and they are ligands of extra- or intra-cellular receptors in many tissues, including the brain, adipose tissue, and some others (7, 8). Conversely, metabolites like 3HK or 3-hydroxyanthranilic acid (3HAA) in some experimental conditions have been shown to induce oxidative stress, but some authors have also demonstrated that they can act oppositely (9, 10). Thus, any intervention that can restore proper KYN metabolism can be of value in the disease’s prevention. Research indicates that these include an exercise demonstrated to induce positive changes in Trp metabolites. For example, regular exercise training on a running wheel has been shown to prevent the stress-induced increase in serum KYN concentration, leading to lower stress perception in the mouse model (11). The changes have been linked with an exercise-induced increase in the activity of kynurenine aminotransferases (KATs) in skeletal muscle, an enzyme that converts KYN to a KYNA metabolite that does not penetrate the blood-brain barrier (BBB) (11). Conversely, both endurance and acute exercise decreased serum Trp concentration and increased both KYN and KYNA levels (12, 13). Interestingly, the effects of acute exercise on the serum level of KP metabolites have been modified by vitamin D supplementation in ultramarathon runners (12). Fasting, besides physical activity, is often used as an intervention with a potential pro-health effect (14). Besides, there is some data showing that these beneficial effects of fasting could also be related to changes in Trp metabolism. For example, 2 days of fasting provide a higher level of picolinic acid (PA) serum concentration, known to have neuroprotective properties (15). From a metabolic perspective, there are some similarities between exercise and fasting (16). In both cases, increased catabolism of stored glycogen and fat is observed, as well as the formation of ketone bodies, activation of intracellular stress signalling, and some others (16). What is more, studies on animals have shown that fasting like an exercise induces an increase in peroxisome proliferator-activated receptor-γ coactivator (PGC)-1α, an expression that is known to stimulate mitochondrial biogenesis, activate genes of gluconeogenesis, β-oxidation, ketogenesis, and increase the expression of KATs (11, 17). Therefore, the purpose of the present study was to investigate whether an 8-day fast applied by a group of healthy men affects the serum concentration of KP metabolites. In addition, we assessed how this fasting period modifies KP metabolites in response to a single bout of exercise of gradually increasing intensity performed before and after the 8-day fast.

## Results

### Effect of fasting on body composition and power output

The 8-day fasting period induced significant changes in the body composition of subjects in the intervention group. Both body weight (BW) and body mass index (BMI) dropped significantly. Also, fat tissue, measured in kilogrammes and as a percentage of BW, was significantly reduced. Notably, the total body water and fat-free mass, expressed as a percentage of total body mass, did not decline during the analysed period. All results are presented in **Table 1**. No changes were recorded in BW (86.82 ± 16.06), BMI (26.78 ± 4.41), or fat tissue mass (21.07 ± 6.09) in the control group. The maximal power output (W) in the study group before intervention was assessed at 204 ± 39.5, while the maximal relative power output (W/kg) was assessed at 2.51 ± 0.43, and after intervention at 183.33 ± 35 and 2.44 ± 0.59, respectively. These differences were statistically insignificant (p = 0.24 and p = 0.78).

**Table 1:** Anthropometric and physiological characteristics of the study group.

Variable	I	II	Δ	CI lower	CI upper	p
**BW [kg]**	84.19 ± 9.49	78.52 ± 9.67	-5.67	-6.96	-4.37	**<0.01**
**Fat [%]**	20.68 ± 3.69	19.67 ± 4.16	-1.01	-1.62	-0.40	**0.01**
**Fat [kg]**	17.68 ± 4.76	15.72 ± 4.79	-1.96	-2.39	-1.52	**<0.01**
**FFM [%]**	79.32 ± 3.7	80.01 ± 3.81	0.70	-0.11	1.51	0.08
**FFM [kg]**	66.51 ± 5.31	62.6 ± 6.04	-3.91	-5.14	-2.68	**<0.01**
**TBW [%]**	58.08 ± 2.72	58.82 ± 3.09	0.75	0.26	1.23	**0.01**
**TBW [kg]**	48.70 ± 3.88	45.98 ± 4.09	-2.72	-3.60	-1.84	**<0.01**
**BMI [kg/m^2^]**	26.35 ± 2.96	24.57 ± 2.94	-1.78	-2.20	-1.37	**<0.01**

Values are means ± SD. I, after overnight fasting; II, after 8 days of fasting; CI – 95% confidence interval; BW, body weight; Fat, body fat; FFM, fat-free mass; TBW, total body water; BMI, body mass index. Significant changes were highlighted in bold, p < 0.05.

### The metabolic response to the 8-day fasting

**Figure 1:**
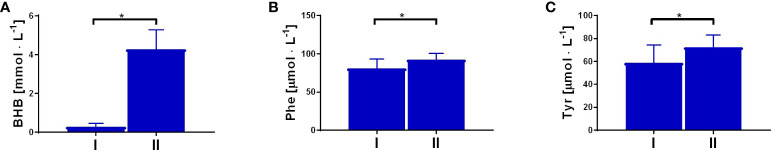
Changes in resting concentration of β-hydroxybutyrate (BHB), p<0.01 **(A)**; phenylalanine (Phe), p=0.01 **(B)** and tyrosine (Tyr), p=0.01 **(C)**, measured before (I) and post the intervention (II). Significant changes were marked with *, p<0.05.

The serum concentration of the ketone body β-hydroxybutyrate, a marker of fasting and carbohydrate dietary restriction (18), increased significantly (**Figure 1A**) in the intervention group. The comparison of KP metabolites in response to the 8-day fasting intervention is presented in **Table 2**. The fasting procedure caused a significant increase in the following KP molecules: 3HK (127.7%), KYNA (159.8%), PA (419.4%), and XA (247.4%). Increased ratios of KYNA/KYN and PA/QA, reflecting kynurenine 3-monooxygenase (KMO) and aminocarboxymuconate semialdehyde decarboxylase (ACMSD) enzyme activity, were also noted. It was also revealed that the ratio values of neuroprotective to neurotoxic KP metabolites, as follows: KYNA/3HK, KYNA/QA, PA/QA, as well as (XA + PA)/(KYN + 3HK), significantly increased in the periphery after the 8-day fast. Additionally, the fasting intervention led to an increase in the serum levels of phenylalanine (Phe) and tyrosine (Tyr), suggesting enhanced catabolism of body proteins (**Figures 1B, C**). The ratio of Phe/Tyr was also calculated, and there were no significant changes in response to the fasting period. A similar tendency was recorded in the control group (**Table 3**).

**Table 2:** Effect of 8-day fasting on KYN metabolites in the intervention group.

Variables	I	II	Δ	CI lower	CI upper	p
**3HAA [nmol/L]**	13.24 ± 5.2	16.7 ± 6.62	4.27	-1.42	9.96	0.12
**3HK [nmol/L]**	95.08 ± 30.32	121.38 ± 22.66	26.30	4.16	48.44	**0.03**
**KYN [µmol/L]**	3.61 ± 1.20	3.33 ± 0.49	-0.28	-1.140	0.586	0.49
**KYNA [nmol/L]**	36.54 ± 7.94	58.39 ± 14.95	21.85	9.67	34.03	**<0.01**
**PA [nmol/L]**	37.62 ± 14.21	157.78 ± 57.47	120.17	79.96	160.37	**<0.01**
**QA [nmol/L]**	343.07 ± 113.5	342.39 ± 88.35	-0.68	-63.82	62.47	0.98
**XA [nmol/L]**	12.43 ± 3.9	30.75 ± 9.95	18.32	10.66	25.97	**<0.01**
**Trp [µmol/L]**	48.36 ± 10.86	46.44 ± 8.57	-1.92	-11.04	7.21	0.65
**KYN/Trp**	15.55 ± 3.77	15.14 ± 2.08	-0.41	-2.97	2.15	0.73
**KYNA/3HK**	0.34 ± 0.11	0.41 ± 0.08	0.07	0.01	0.13	**0.03**
**KYNA/KYN**	0.01 ± 0	0.02 ± 0	0.01	0.00	0.01	**0.01**
**KYNA/QA**	0.13 ± 0.04	0.21 ± 0.08	0.08	0.03	0.13	**0.01**
**PA/QA**	0.09 ± 0.05	0.38 ± 0.19	0.28	0.15	0.42	**<0.01**
**(XA+PA)/(KYN+3HK)**	0.01 ± 0.01	0.04 ± 0.01	0.03	0.02	0.04	**<0.01**

Values are means ± SD. I, after overnight fasting; II, after 8 days of fasting; CI, 95% confidence interval; 3HAA, 3-hydroxyanthranilic acid, 3HK, 3-hydroxy-L-kynurenine, KYN, kynurenine; KYNA, kynurenic acid; PA, picolinic acid; QA, quinolinic acid; XA, xanthurenic acid; Trp, tryptophan; Significant changes were highlighted in bold, p < 0.05.

**Table 3:** KYN metabolites at rest in the control group.

Variables	I	II	Δ	CI lower	CI upper	p
**3HAA [nmol/L]**	56.34 ± 44.56	56.13 ± 22.09	-0.22	-16.36	15.93	0.63
**3HK [nmol/L]**	18 ± 6.67	18.21 ± 6.29	0.21	-1.71	2.14	0.83
**KYN [µmol/L]**	2.75 ± 0.61	2.71 ± 0.58	-0.05	-0.3	0.2	0.71
**KYNA [nmol/L]**	52.94 ± 20.78	50.47 ± 16.89	-2.47	-9.26	4.31	0.46
**PA [nmol/L]**	49.39 ± 20.64	51.35 ± 21.05	1.97	-7.46	11.39	0.97
**QA [nmol/L]**	255.52 ± 145.29	249.08 ± 111.85	-6.44	-40.01	27.13	0.70
**XA [nmol/L]**	21.03 ± 10.37	20.33 ± 8.71	-0.70	-4.58	3.18	0.71
**Trp [µmol/L]**	66.45 ± 17.42	65.21 ± 10.73	-1.24	-6.83	4.34	0.69
**KYN/Trp**	43.67 ± 13.2	42.79 ± 11.71	-0.88	-5.06	3.3	0.66
**KYNA/3HK**	3.27 ± 1.95	2.98 ± 1.11	-0.29	-1.02	0.45	0.88
**KYNA/KYN**	0.01 ± 0.01	0.02 ± 0.01	0.00	0.00	0.00	0.71
**KYNA/QA**	0.24 ± 0.09	0.23 ± 0.11	-0.01	-0.04	0.02	0.54
**PA/QA**	0.22 ± 0.08	0.23 ± 0.10	0.01	-0.01	0.03	0.44
**(XA+PA)/(KYN+3HK)**	0.03 ± 0.01	0.03 ± 0.01	0.00	0.00	0.01	0.91
**Tyr [µmol/L]**	71.01 ± 15.67	77.81 ± 23.12	6.80	-1.35	14.95	0.10
**Phe [µmol/L]**	83.59 ± 13.26	94.73 ± 23.40	11.13	1.08	21.19	0.03
**Phe/Tyr [µmol/L]**	1.21 ± 0.22	1.28 ± 0.32	0.07	-0.05	0.18	0.54

Values are means ± SD. I, before; II, after control time; 3HAA, 3-hydroxyanthranilic acid; 3HK, 3-hydroxy-L-kynurenine; KYN, kynurenine; KYNA, kynurenic acid; PA, picolinic acid; QA, quinolinic acid; XA, xanthurenic acid; TRP, tryptophan; Tyr, tyrosine; Phe, phenylalanine; p<0.05.

### The 8-day fast modified the KP response to a single bout of exercise

**Figure 2:**
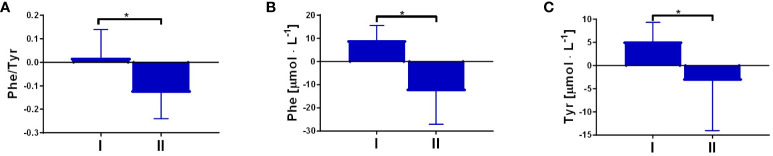
The response to a single bout of exercise performed before and post-intervention in the ratio of Phe/Tyr, p = 0.02 **(A)**; and the concentration of phenylalanine (Phe), p < 0.01 **(B)**; and tyrosine (Tyr), p = 0.04 **(C)**. Significant changes were marked with *, p<0.05.

To evaluate the effect of fasting and a single bout of exercise on Trp metabolism, serum samples were collected at rest and 1 h post-exercise, once overnight (Δ1), and again after an 8-day fasting period (Δ2). Data showed that the single bout of exercise performed after the 8-day fasting intervention significantly decreased serum levels of 3HAA (Δ2 = -2.93 ± 5.31) and PA (Δ2 = -12.42 ± 24.09) metabolites compared to results obtained after short overnight fasting (Δ1 = 2.72 ± 4.53 and 5.67 ± 6.9, respectively). Furthermore, the ratios of KYNA/KYN (Δ2 = 4.79 ± 3.59, p < 0.05) and KYNA/QA (Δ2 = 38.99 ± 23.88, p < 0.05) significantly increased after the acute exercise and fasting intervention compared to values obtained for the first time post-overnight fast (Δ1 = -0.86 ± 1.89 and 7.11 ± 14.72, respectively). Interestingly, a significant increase in Phe (Δ1 = 9.22 ± 6.22, p < 0.05) and Tyr (Δ1 = 5.27 ± 4.07, p < 0.05) serum levels was observed after the exercise test before the 8-day fast. However, a decline in their levels was noted after the fasting protocol implementation (Δ2 = -12.85 ± 14.35, p < 0.05 and Δ2 = -3.3 ± 10.76, p < 0.05, respectively) (**Figures 2B, C**). The Phe/Tyr ratio showed a significant decline as a result of the exercise test performed after the fast (Δ2 = -0.13) (**Figure 2A**). As Tyr is a known precursor of dopamine, these results may reflect favourable conditions for its production as a result of the effect of physical exercise performed after an 8-day fast. All results are presented in **Table 4** and in **Figure 2**.

**Table 4:** Effects of a single bout of exercise on KYN metabolites before and after an 8-day fasting period.

Variables	Δ1	Δ2	Diff	ANOVA
**3HAA [nmol/L]**	2.72 ± 4.53	-2.93 ± 5.31	-6.07	**0.03**
**3HK [nmol/L]**	6.54 ± 14.49	5 ± 12.1	-1.37	0.81
**KYN [µmol/L]**	0.45 ± 0.45	0.03 ± 1.20	-0.43	0.32
**KYNA [nmol/L]**	3.9 ± 5.02	12.43 ± 12.66	8.12	0.07
**PA [nmol/L]**	5.67 ± 6.9	-12.42 ± 24.09	-17.60	**0.04**
**QA [nmol/L]**	20.24 ± 40.34	3.81 ± 65.75	-12.13	0.52
**XA [nmol/L]**	2.34 ± 1.63	2.02 ± 3.6	-0.42	0.80
**Trp [µmol/L]**	4.54 ± 7.23	-3.3 ± 10.76	-7.50	0.08
**KYN/Trp**	0.02 ± 0.24	0.05 ± 0.13	-0.03	0.38
**KYNA/KYN**	-0.86 ± 1.89	4.79 ± 3.59*	5.83	**< 0.01**
**KYNA/QA**	7.11 ± 14.72	38.99 ± 23.88*	30.68	**< 0.01**
**PA/QA**	9.61 ± 26.41	-40.04 ± 77.07	-45.98	0.20
**KYNA/3HK**	2.91 ± 63.25	79.50 ± 103.38	81.72	0.07
**(XA+PA)/(KYN+3HK)**	2.29 ± 3.92	0.19 ± 2.18	-2.80	0.76

Values are means ± SD. Δ1, change after the first single bout of exercise; Δ2, change after the last single bout of exercise; Diff: difference between changes after the first and last single bout of exercise; 3HAA, 3-hydroxyanthranilic acid; 3HK, 3-hydroxy-L-kynurenine; KYN, kynurenine; KYNA, kynurenic acid; PA, picolinic acid; QA, quinolinic acid; XA, xanthurenic acid; Trp,tryptophan; Significant changes were highlighted in bold or marked with *, p < 0.05.

## Discussion

In the present study, we demonstrated that 8 days of fasting significantly modified the resting levels of serum Trp metabolites. The effects of acute exercise on kynurenines were distinct after the interventional period of fasting compared to only overnight fasting. Several reports have investigated the effects of different training protocols on kynurenine profiles (12, 13, 19, 20) among aged subjects and athletes, assessing kynurenine metabolites in plasma (21, 22) or human sweat (23). While the effects of exercise are deeply investigated, it is known that other factors, such as vitamin D (12) and omega-3 fatty acids (24), can also modify the exercise-induced response. However, data regarding the effects of long-term fasting on exercise-induced kynurenine profiles remain limited.

As mentioned above, fasting can induce some extended changes similar to those observed after exercise. For example, during both fasting and prolonged exercise, carbohydrate availability is limited, leading to lipid metabolism becoming the primary energy source (16). Consequently, ketone bodies are formed, an effect observed in this study. Skeletal muscle proteins can be an essential source of amino acids, which can serve as energy substrates, especially when carbohydrate availability is limited. These amino acids can also be used for the biosynthesis of metabolites like nitric oxide, kynurenines, and hormones. There are additional adaptive changes induced by both fasting and exercise training. One notable change is the activation of PGC-1α, a transcriptional factor that induces the expression of KAT enzymes, which catalyse the formation of KYNA from KYN (8, 17). Such a change was noted after the 8-day fasting period. Furthermore, the tendency for a higher increase in KYNA in response to acute exercise in subjects after the 8-day fasting period, compared to exercise performed only after overnight fasting, indicates that fasting induced a shift in the KYN pathway, possibly by enhancing KAT activity. Interestingly, the changes in KYN after acute exercise did not reach statistical significance. Similarly, previous studies have shown that plasma KYN levels do not alter significantly, while KYNA (19) levels increase after endurance exercise. In the present study, we demonstrated that 8 days of fasting significantly modified the resting levels of serum Trp metabolites.

Considering that fasting was accompanied by an increase in blood KYNA, it can be speculated that this conversion prevents KYN accumulation. An increase in serum KYNA is very desirable and can indicate improved skeletal muscle metabolism. KYNA, besides its function in the CNS, can modify metabolism in the periphery as an agonist of the G protein-coupled receptor 35 (GPR35) or as an antagonist of the N-methyl-D-aspartate receptor (NMDAR). KYNA has been demonstrated to reduce adipocyte insulin resistance and inflammation (25) or increase the browning of adipocytes (7). KYN can also be converted into 3HK in a reaction catalysed by kynurenine-3-hydroxylase, and 3HK can then be converted into XA (1). Our results demonstrated no change in XA after exercise, but fasting doubled its concentration. This is a significant effect of fasting, as XA is considered a neuroprotective compound and is known to stimulate dopamine release in the cortex and striatum (20). 3HK is further converted into 3HAA in a reaction catalysed by kynureninase, which is then transformed into α-amino-β-carboxymuconate-ϵ-semialdehyde (ACMS). ACMS is non-enzymatically converted into QA or, in a reaction catalysed by picolinic carboxylase, into PA (26). Our results suggest that fasting leads to increased formation of neuroprotective PA but not neurotoxic QA. This is a promising observation, as PA, contrary to QA, penetrates the blood-brain barrier (BBB). Thus, an increase of around 420% in PA after fasting can be expected to lead to an increase in its concentration in the central nervous system (27). Furthermore, among the KP metabolites, XA, KYN, and 3HK easily penetrate the BBB (27). Interestingly, the ratio of neuroprotective (XA + PA) to neurotoxic KP metabolites (KYN + 3HK) (1) significantly increases after fasting. These results suggest that fasting may exert neuroprotective effects. Conversely, an increase in Phe might have some negative effects (28). During fasting, serum Trp is taken up by different tissues and metabolised; hence, its concentration should decrease. Indeed, serum metabolites of Trp like KYNA, XA, and PA increased significantly; however, Trp concentration remained unchanged post-fasting. To understand this phenomenon, the aromatic amino acids Phe and Tyr were investigated. The concentration of these two amino acids could indicate the balance between protein synthesis and degradation (29). Since fasting has been shown to stimulate skeletal muscle protein degradation, some amino acids are released into the blood and can be used, for example, as substrates for gluconeogenesis to increase glucose formation. While amino acids like Phe are poorly metabolised, fasting causes their net release to be elevated in skeletal muscle (29). The observed increase in blood Phe and Tyr after 8-day fasting suggests increased proteolysis of skeletal muscle and possibly other tissues (30, 31). According to Palmer et al., these changes have already been initiated in the gluconeogenic phase of fasting, characterised by an increased concentration of traditional glucogenic amino acids (32). Augmented proteolysis can also lead to an increase in the blood supply of Trp, and it cannot be excluded that the augmented formation of some Trp metabolites results from such a process. For example, serum Trp decreases and KYN increases after an ultramarathon run, especially in athletes supplemented with vitamin D (12). It has been suggested that this is due to the anti-proteolytic function of vitamin D, which was demonstrated to reduce atrogin-1 levels in human skeletal muscle (33). Atrogin-1 stimulates skeletal muscle atrophy, and its expression increases following endurance exercise (34).

**Figure 3:**
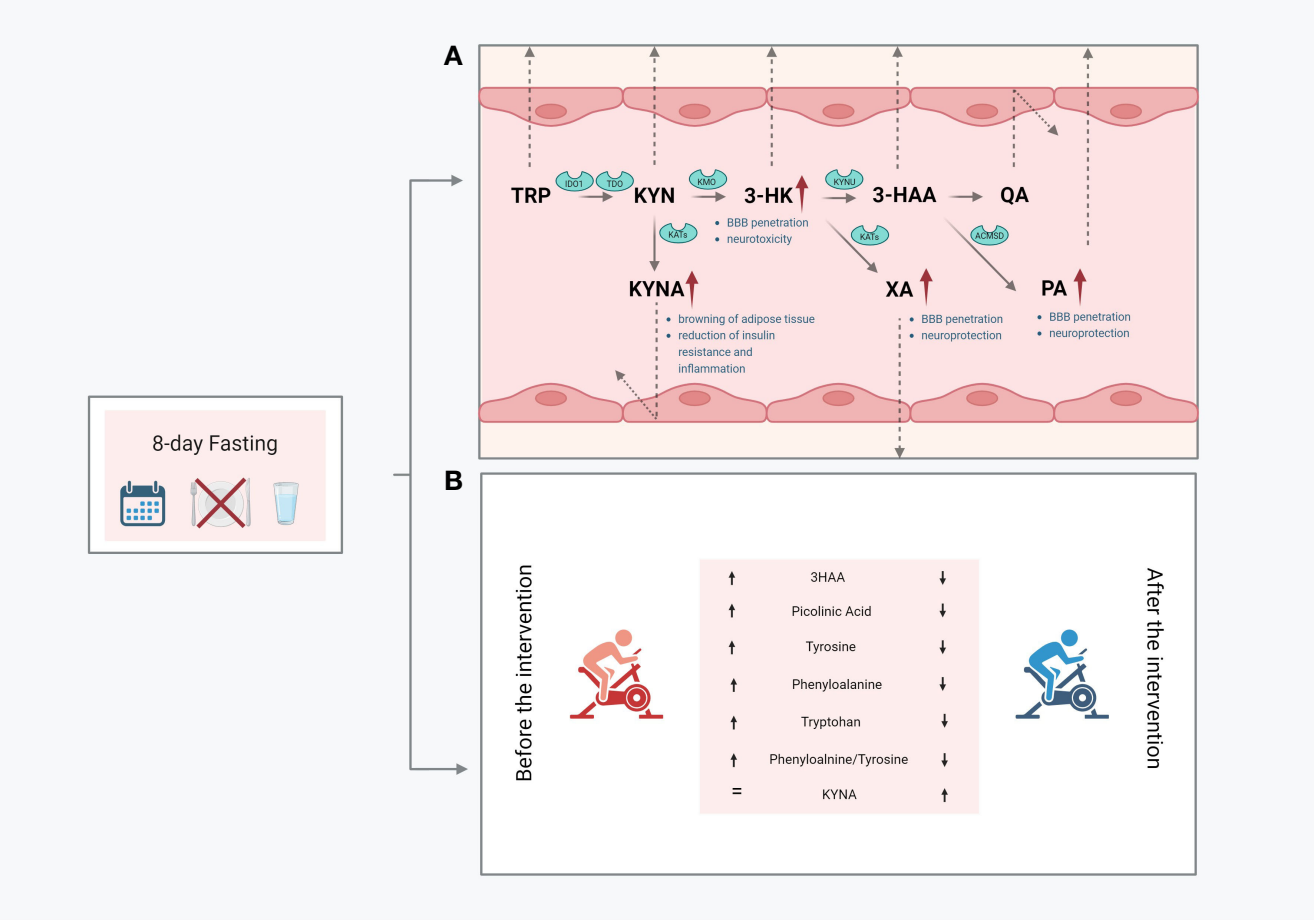
Graphical summary of the obtained outcomes: the impact of resting values of KP metabolites **(A)**; changes in response to a single bout of exercise performed before and after the intervention **(B)**; 3-hydroxyanthranilic acid, 3HK, 3-hydroxy-L-kynurenine, KYN, kynurenine; KYNA, kynurenic acid; PA, picolinic acid; QA, quinolinic acid; XA, xanthurenic acid; Trp, tryptophan.

In conclusion, we demonstrated that fasting significantly modified the serum concentration of metabolites in the KYN pathway. The observed changes, mainly manifested by increased serum concentrations of PA, XA, and KYNA, can be considered desirable. Both PA and XA can penetrate the BBB and are known to exert neuroprotective actions. Conversely, serum KYNA is expected to act on the periphery of the central nervous system and can initiate several beneficial changes, including the browning of adipose tissue and the reduction of insulin resistance and inflammation (**Figure 3**). These findings suggest that the widely reported health-promoting effects of fasting are also associated with metabolic changes in the KYN pathway.

### Study limitations

The main limitations of this study include the small number of participants, the lack of gender diversity, and the absence of muscle biopsy sampling. Despite efforts, maintaining such a prolonged fasting period presented a significant challenge, leading to the withdrawal of four participants. However, it is worth noting that our study group is highly homogeneous and disciplined, which enhances the credibility of the results. Additionally, participants are experienced in fasting practices, reducing the risk of experimental failure, particularly as prolonged fasting is not a typical routine for European Caucasians. Notwithstanding, these limitations require some caution in interpreting the results. For instance, baseline level differences were observed in some indicators between participants and the control group, who lacked experience with fasting. In summary, future studies should address these limitations by including larger participant cohorts and incorporating additional time points for blood collection and muscle biopsies.

## Materials and methods

### Ethics

All participants enrolled in the research had prior experience fasting before the intervention. The study protocol was tailored to accommodate the fasting regimen of the recruited volunteers. Each participant received detailed information regarding the aims, methods, and potential risks associated with the study and provided written consent. Ethical approval was granted by the Committee for Ethics in Scientific Research of Jan Długosz University in Czestochowa (Poland; KE-0/1/2019; 5 March 2019), adhering to the principles outlined in the Declaration of Helsinki—Ethical Principles for Medical Research Involving Human Subjects. To ensure safety, all subjects were under medical supervision for 3 days before the examination, throughout the 8-day fasting period, and for 3 days after the completion of the study.

### Characteristics of the subjects

A total of 14 healthy men were initially recruited for the study group, although only 10 individuals completed the intervention (average age: 54.40 ± 13.16 years). Additionally, 10 healthy individuals were included in our research as a control group (average age: 36.42 ± 6.65 years). No contraindications between physical exercise and fasting interventions were identified in participants during the medical examination. Participants reported previous fasting experiences ranging from 3 days to 42 days. Other inclusion criteria included an age between 30 years and 70 years, body weight between 60 kg and 100 kg, body mass index (BMI) between 20 kg/m² and 29.9 kg/m², systolic blood pressure ranging from 100 mmHg to 140 mmHg, and diastolic blood pressure ranging from 60 mmHg to 90 mmHg. Exclusion criteria comprised any chronic diseases, smoking tobacco products, medication intake, the use of potent stimulants and psychoactive substances, and failure to complete the training protocol. The study group was classified as having daily moderate-intensity physical activity, although the level of physical activity among subjects was not controlled.

### Study intervention

**Figure 4:**
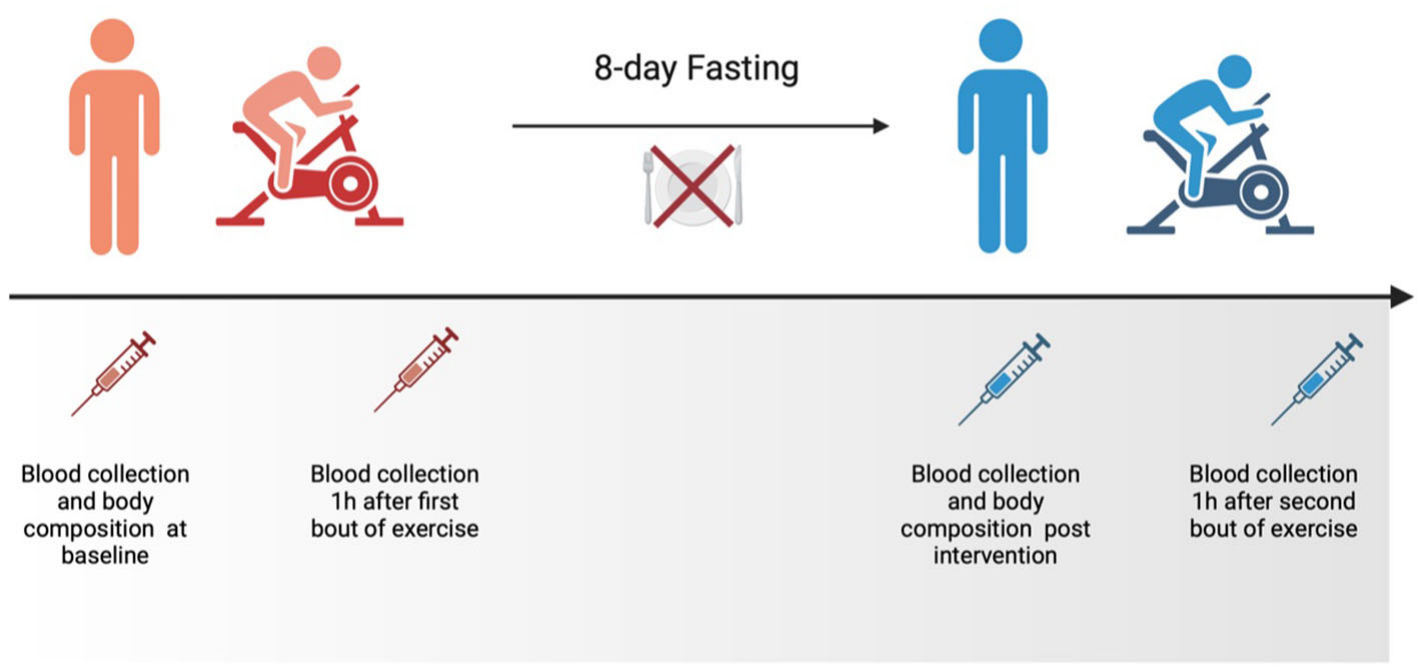
The schedule of the experiment.

During the 8-day intervention, volunteers reported consuming only water (containing an average amount of ions). Additionally, they were instructed to engage in regular, daily moderate-intensity physical activity and maintain their usual daily habits. Consumption of any food or beverage containing calories was prohibited during the examination period. No side effects of fasting were documented. The effect of fasting on physical performance and KP metabolites was evaluated at baseline and after 8 days of the intervention (**Figure 4**). No alcohol or medication intake, as well as exercise performance, was allowed for 2 days before the start of the project.

### Anthropometric measurements

Anthropometric measurements of body composition, including fat tissue expressed in kilogrammes and percentage of fat-free mass (FFM), were conducted at baseline and after 8 days of intervention using the bio-electric impedance method (TBF 300A body composition analyser, Tanita, Amsterdam, Netherlands). The body mass index (BMI) was calculated using the formula: weight (kg)/height (m)^2^. The somatic body parameters were measured in the morning on an empty stomach after a 12-h fast, and measurements were taken after a night’s rest.

### Exercise test

To evaluate how 8 days of fasting might modify exercise response, we decided to employ a commonly used protocol for cardiorespiratory fitness assessment. This protocol, performed at two time points (before and after 8 days of fasting), involved an exercise test with progressively increasing intensity. It entailed 30W increments every 3 min until exhaustion and was conducted on the Excalibur Sport cycle ergometer (Lode B.V., Groningen, The Netherlands). The baseline physical workload was set at 60W, with the work rate escalating incrementally. Maximal power output, both absolute and relative, was measured in Watts and Watts per kilogramme, respectively. The exercise was halted under certain conditions: when the participant’s oxygen uptake reached a maximum peak or declined, when the heart rate failed to increase, stabilise at its maximum level, or decrease, or when the pedalling rhythm could not be maintained.

### Sample preparation

Blood samples were collected before and after the intervention between 7:00 a.m. and 8:00 a.m. under fasting conditions. The blood was drawn from the antecubital vein into vacutainer tubes (Becton Dickinson, USA) by a professional nurse and then centrifuged at 2000 g for 10 min at 4°C to obtain serum, which was subsequently stored at -80°C until assayed. The sample preparation protocol involved serum protein precipitation, followed by a derivatisation step. Upon quick thawing, 50 µL of serum was transferred to a 1 mL deep-well plate (propylene 96-well plate). The serum was then precipitated using 250 µL of the precipitation reagent, comprising internal standards dissolved in acetonitrile. Samples were stirred for 30 min at 1100 rpm and centrifuged for 10 min at 3000 rpm. Subsequently, 50 µL of the supernatant was gently aspirated and transferred to a new 96-well polypropylene plate (with a working capacity of 300 µL), where it was dried. A 3M hydrogen chloride 1-butanol solution (Merck KGaA, Darmstadt, Germany) was added for derivatisation. The dried samples were dissolved in 100 µL of a 0.1% aqueous solution of formic acid (VWR, Radnor, PA, USA) and then injected into a chromatographic system.

### LC-MS/MS analysis

The following KYN metabolites were quantitatively analysed in serum using high-performance liquid chromatography with tandem mass spectrometry (UFLC system, Shimadzu, Kioto, Japan, coupled with QTRAP^®^5500+, Sciex, Framingham, MA, USA): 3HAA, 3HK, KYNA, PA, QA, and XA. For chromatographic separation, a Zorbax Eclipse XDB-C18 column (50 mm × 4.6 mm, 1.7 μm; Agilent, Santa Clara, CA, USA) was utilised. The mobile phases consisted of acetonitrile and water (VWR, Radnor, PA, USA) with 0.1% formic acid. Only LC-MS-grade solvents were employed. Samples were injected into the chromatographic system in a volume of 10 µL. The analyte separation run time was 6 min, conducted at a flow rate of 0.8 ml/min. Initially, the analysis was performed using the isotopic dilution method, known for its resistance to instrument-sensitivity-dependent variability. This method normalises results by the area of the isotopic reference standard for each analyte. Instrument drift was controlled at two independent levels. The variability of isotopic standards within the entire batch (all samples) was analysed, along with blanks and two quality control samples in each measurement batch. A blank sample (reagent blank) assessed changes in background signals and false positives, while quality control samples, consisting of two patient samples with different concentrations of analytes, evaluated batch-to-batch variability.

The measurement was conducted using multiple reaction monitoring (MRM) in positive electrospray ionisation mode. Concentration ranges for KYN metabolites were as follows: 10 ng/ml–1000 ng/ml for KYN; 1 ng/ml–100 ng/ml for 3HAA, 3HK, and KYNA; 5 ng/ml–500 ng/ml for QA; 0.5 ng/ml–50 ng/ml for PA and XA. Raw data were collected using Analyst^®^ software, and MultiQuant^®^ (Sciex, Framingham, MA, USA) was employed for data processing and quantification.

### Statistical analysis

Statistica 13.1 software was employed for statistical analysis, with results presented as mean ± standard deviation (SD). The Shapiro-Wilk test assessed the homogeneity of dispersion from a normal distribution, while the Brown-Forsythe test evaluated the homogeneity of variance. For homogenous results, a paired t-test analysis was conducted to identify significantly different results, whereas for heterogeneous results, the Wilcoxon signed-rank test was used. Furthermore, the difference between measurements was calculated (Δ) with 95% confidence intervals (CI). Additionally, the response to a single bout of training before and after fasting was investigated. For homogenous results, analysis of variance (ANOVA) for repeated measures, followed by *post-hoc* Tukey’s test for equal sample sizes, was employed to identify significantly different results. For heterogeneous results, ANOVA, Friedman’s test, and Dunn-Bonferroni *post-hoc* tests were utilised. The significance level was set at p < 0.05.

## Data availability statement

The raw data supporting the conclusions of this article will be made available by the authors, without undue reservation.

## Ethics statement

The studies involving humans were approved by Committee for Ethics in Scientific Research of Jan Długosz University in Czestochowa (Poland; KE-0/1/2019; 5 March 2019). The studies were conducted in accordance with the local legislation and institutional requirements. The participants provided their written informed consent to participate in this study.

## Author contributions

UJ: Conceptualization, Data curation, Investigation, Methodology, Writing – original draft, Writing – review & editing. JR: Investigation, Methodology, Writing – review & editing. JAK: Conceptualization, Data curation, Software, Writing – review & editing. MŻ: Investigation, Methodology, Writing – review & editing. KP: Conceptualization, Investigation, Methodology, Writing – review & editing. EZ: Investigation, Methodology, Writing – review & editing. IC: Investigation, Methodology, Software, Writing – review & editing. JA: Conceptualization, Funding acquisition, Methodology, Supervision, Writing – original draft, Writing – review & editing. AB: Conceptualization, Investigation, Methodology, Supervision, Writing – review & editing.
